# T cell senescence and CAR-T cell exhaustion in hematological malignancies

**DOI:** 10.1186/s13045-018-0629-x

**Published:** 2018-07-04

**Authors:** Dimitri Kasakovski, Ling Xu, Yangqiu Li

**Affiliations:** 1 Key Laboratory for Regenerative Medicine of Ministry of Education, Institute of Hematology, School of Medicine, Jinan University, Guangzhou, 510632 China; 2 Department of Anatomy and Molecular Embryology, Institute of Anatomy, Ruhr-University Bochum, 44801 Bochum, Germany; 3 Department of Hematology, First Affiliated Hospital, School of Medicine, Jinan University, No. 601 West of Huangpu Avenue, Guangzhou, 510632 China

**Keywords:** T cells, Senescence, Hematological malignancy, T cell activation, CAR-T cells

## Abstract

T cell senescence has been recognized to play an immunosuppressive role in the aging population and cancer patients. Strategies dedicated to preventing or reversing replicative and premature T cell senescence are required to increase the lifespan of human beings and to reduce the morbidity from cancer. In addition, overcoming the T cell terminal differentiation or senescence from lymphoma and leukemia patients is a promising approach to enhance the effectiveness of adoptive cellular immunotherapy (ACT). Chimeric antigen receptor T (CAR-T) cell and T cell receptor-engineered T (TCR-T) cell therapy highly rely on functionally active T cells. However, the mechanisms which drive T cell senescence remain unclear and controversial. In this review, we describe recent progress for restoration of T cell homeostasis from age-related senescence as well as recovery of T cell activation in hematological malignancies.

## Background

The immune system plays a crucial role in the protection and fight against hematological malignancies and cancer [1–3]. Impairment of the immune system due to a decrease in immunological diversity of naïve T cells and an increasing number of senescent T cells with age leads to a higher susceptibility to disease and potentially promotes progression of malignant tumor in elderly [4, 5]. Moreover, human cytomegalovirus (HCMV) persistence occurs upon repeated T cell activation due to chronic infections with CMV and is considered a driver of immune senescence in humans, starting from puberty after thymic involution [6]. Nevertheless, cellular senescence can also act as a protective mechanism of the immune system against cancer by deactivating T cells which show excessive or aberrant proliferation [7–9]. T cell senescence is triggered in a variety of biological processes including tumor prevention, immune response to infections, and aging. It leads to distinctive phenotypic and functional alteration and can be caused by tumor-associated stresses, telomere damage, and regulatory T (Treg) cells [4, 10]. Here, we summarize recent findings of the role of senescent T cells in hematological malignancies as well as possibilities to restore function of senescent and exhausted T cells for immunotherapies, such as CAR-T cell therapy.

### Discovery and concept of T cell senescence

Cellular immune senescence was firstly described in the late 70s and was mainly focused on age-dependent changes in macrophages and lymphocytes in mice. Previous findings show less influence of aging on macrophages, while lymphocytes show considerable changes during aging. Especially, T cells due to their relatively long lifespan of 4–6 months have time to mature and express different functions with age [11, 12]. Recently, immunosenescence and T cell senescence are described as the degeneration of innate and adaptive immunity and specifically as a depletion of naïve and effector T cells during aging. Nearing the end of their lifespan, T cells can become senescent, characteristically leading to a cell-cycle arrest while staying viable and metabolically active [13]. T cell senescence can be distinguished from T cell anergy and T cell exhaustion which share similar characteristics but have different origins. T cell anergy is a hyporesponsive state in T cells which is triggered by excessive activation of the T cell receptor (TCR) and either strong co-inhibitory molecule signaling or limited presence of concomitant co-stimulation through CD28. T cell exhaustion occurs after repeated activation of T cells during chronic infection or tumor progression. In acutely cleared infections, a part of activated T cells develops into highly functional memory T cells, while in chronic infections and the tumor microenvironment, the persistent activation of T cells can lead to a gradual development into an exhausted phenotype. This phenotype is defined by poor effector function and sustained expression of inhibitory receptors [14]. While both T cell anergy and T cell exhaustion in natural occurrence are considered reversible, T cell senescence until recently was considered irreversible [15–18]. Recent studies challenge this distinction by showing that senescent T cells are in fact able to regain function by inhibiting the p38 mitogen-activated protein kinase (MAPK) pathway and show relationships between T cell exhaustion and senescence [19, 20].

### Mechanisms of T cell senescence

T cell senescence can be triggered by two major cellular mechanisms: replicative and premature senescence. Replicative senescence is the natural age-related process that occurs after several rounds of proliferation leading to a shortening of telomeric ends. The cell is then put into a senescent state to prevent a potential degeneracy into a cancerous cell. The second mechanism is premature senescence which is a telomere-independent senescence induced by outside factors such as cellular stress [21–23]. For example, effector T cells, CD4^+^ helper, and CD8^+^ cytotoxic T cells can be forced by Treg cells into senescence, by inducing DNA damage using metabolic competition during cross-talk [22].

### Biomarkers for T cell senescence and T cell exhaustion

Although in recent years molecular and cellular biomarkers of effector T cell differentiation have been studied extensively, many of the molecular and signaling pathways related to maturation and senescence of effector T cells are still unknown. T cells in replicative senescence tend to lose co-stimulatory molecules such as CD27 and CD28 while expressing killer cell lectin-like receptor subfamily G (KLRG-1) and CD57. Interfering with the ligation of KLRG-1 on T cells has shown enhanced proliferation capability. CD57 was shown to be associated with severe proliferation impairment and thus is considered the most reliable surface marker for T cell senescence. Furthermore, G1-regulating proteins such as p15, p16, and p21 which are involved in cell cycle regulation and are associated with cellular stress response are upregulated in senescent T cells, with evidence of increased levels of bound p16/Cdk6 and p21/WAF, downregulation of Cdk2 and cyclinD3 expression, and decreased Cdk2 and Cdk6 kinase activity. These molecules inhibit the transition from G1 to S phase forcing cells into a replicative senescence [24–29]. Additionally, CD27 and CD28 downregulation is associated with loss of human telomerase RNA component (hTERC) expression, leading to a decrease in telomerase activity and subsequent impaired buildup of telomeric ends [29, 30]. In a recent study, the T cell immunoreceptor with Ig and tyrosine-based inhibitory motif (ITIM) domains (TIGIT) was suggested to be a novel T cell senescence marker. TIGIT was shown to be upregulated in CD8^+^ T cells of elderly in comparison to young individuals. Moreover, TIGIT^+^CD8^+^ T cells exhibited a senescence immunophenotype including high expression of KLRG1 and CD57 while retaining cytotoxicity and function, thus linking the mechanisms of T cell senescence to previous findings pertaining to the role of TIGIT in the mechanism of T cell exhaustion [31, 32]. Furthermore, the negative checkpoint receptor TIGIT was described as a novel marker in exhausted CD4+ and CD8+ T cells after HIV infection [33, 34]. Exhausted T cells hierarchically lose the production of IL-2, their high proliferative capacity and ability for ex vivo killing, followed by loss of production of tumor necrosis factor (TNF), and in the last stage, partial or complete loss of the ability to produce large amounts of interferon-γ which ultimately leads to physical deletion. This decline of effector function is accompanied by a progressive loss of CD4+ T cell help and increased expression of inhibitory receptors, e.g., PD1, CTLA4, TIGIT, LAG-3, CD244, CD160, or TIM3 [35, 36].

### T cell senescence progression in aging healthy individuals

**Fig. 1:**
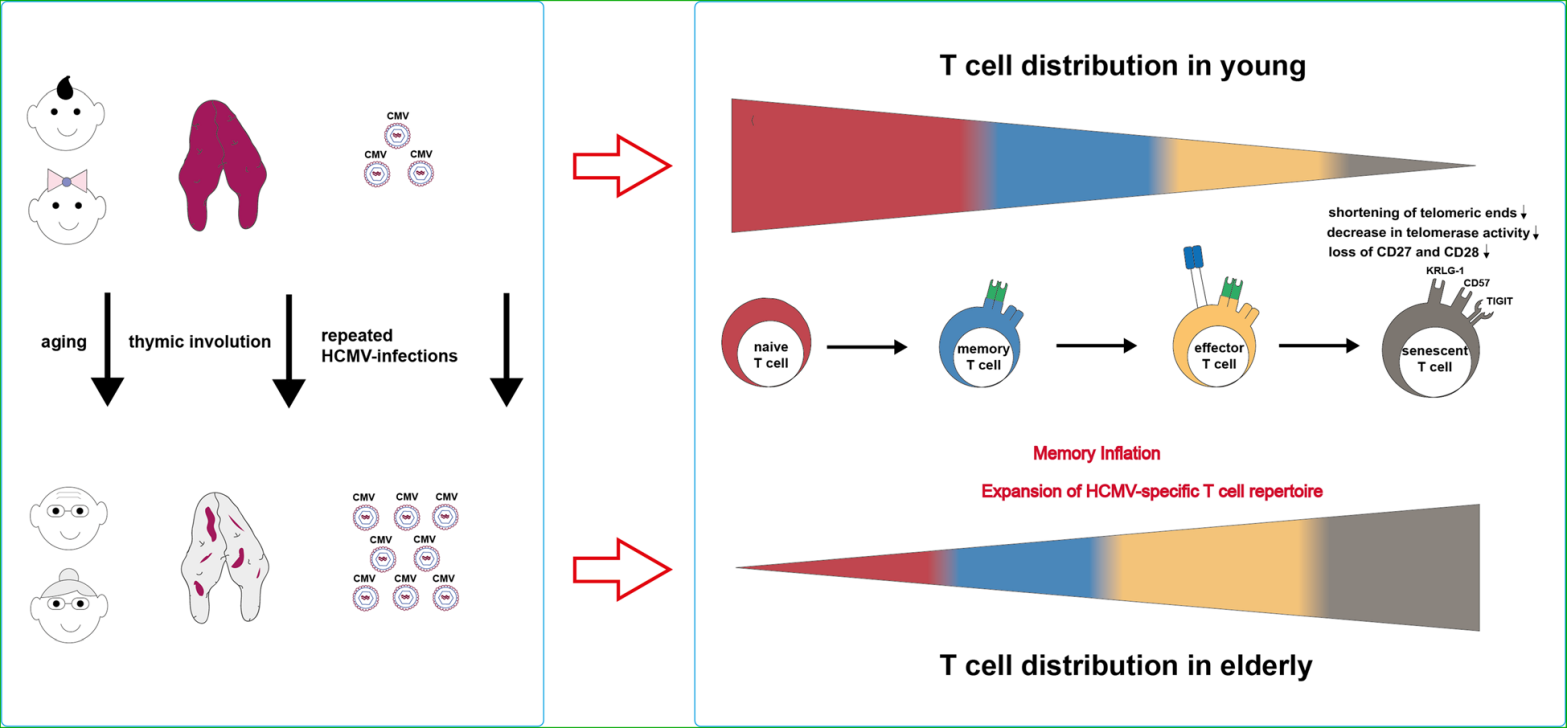
Schematic model of age and HCMV infection related T cell immunosenescence in the peripheral blood of human. Memory inflation due to latent HCMV infections and thymic involution lead to a shift in T cell distribution from mainly naïve and memory T cells towards effector and senescent T cells with progressing age. Senescent T cells are characterized by shortening of telomeric ends, decrease in telomerase activity, and loss of CD27 and CD28 expression. Markers of T cell senescence include KRLG1, CD57, and the recently identified receptor TIGIT

Many countries face demographic changes in their population with an over-proportional increase in the elderly in comparison to the young. T cell senescence impairs life-long immune protection and effective vaccination by limiting variability. T cell composition is shifted from undifferentiated naïve T cells to determined memory T cells and further to senescent T cells [4, 13]. The output of naïve T cells decreases after puberty and thymic involution, leading the remaining naïve T cells to progressively become determined and differentiated during lifetime. While the proportion of naïve T cells decreases in early life, the proportion of differentiated memory T cells increase until it reaches a stable plateau during adulthood. After the age of 65, a shift to senescence and an accumulation of highly differentiated CD28^−^ T cells are observed [37]. This accumulation occurs especially strong with respect to the CD8^+^CD28^−^ T cell subset which expresses enhanced cytotoxicity and regulatory functions while having a shorter replicative lifespan and defective antigen-induced proliferation [28, 37]. There is a growing body of evidence that age-related T cell senescence is not only caused by thymic involution but is also accelerated by memory inflation caused by HCMV infection. HCMV infection is significantly associated with changes in both naïve CD4^+^ T cell composition as well as memory T cells of the CD8^+^ subset. Memory inflation leads to an accumulation of HCMV-specific CD8^+^CD28^−^ T cells which also express typical senescence marker such as KRLG1 and CD57 while remaining highly cytotoxic. This excess expansion of a single HCMV-specific repertoire can occupy up to 50% of the entire CD8^+^ T cell and 30% of CD4^+^ T cell compartment of the peripheral blood in HCMV-infected elderly individuals [38–40]. This might indicate a joint responsibility of age-related and HCMV-related T cell senescence in the impaired immune response to vaccination as well as an increased susceptibility towards disease and hematological malignancies in elderly individuals. Factors which contribute to T cell senescence and altered T cell subset distribution from young to elderly individuals are shown in Fig. 1.

### T cell senescence in hematological malignancies

Malignant tumors utilize many different strategies to evade anti-tumor immunity of the adaptive immune system by creating immunosuppressive microenvironments [39, 40]. Mechanisms of immune evasion include alteration of G1-regulating protein expression, production of suppressive factors like interleukin-10 (IL-10), transforming growth factor beta (TGF-β), and indoleamine-pyrrole 2,3-dioxygenase (IDO) as well as expression of immune inhibitory receptors, e.g., programmed cell death ligand 1 (PD-L1) and recruitment of Treg cells [41–43]. An accumulation of senescent CD8^+^CD28^−^ T cells was observed in several solid tumors, indicating the use of the suppressive activity of senescent T cells as a strategy for immune evasion [44–47]. Tumor-derived cAMP was shown to be responsible for the direct induction of senescence in T cells and is also a key component of the Treg cell mechanism of forcing T cells into senescence [48]. These findings correlate with re-occurring observations of Treg cell accumulations in hematological malignancies such as acute myeloid leukemia (AML), acute lymphoblastic leukemia (ALL), chronic lymphocytic leukemia (CLL), multiple myeloma (MM), and B cell lymphomas [49–52]. Conclusively, reduced Treg cell accumulation significantly prognosticated low relapse risk and leukemia-free survival (LFS) in AML patients [48, 49]. Recently, senescent T cells including clonally expanded CD8^+^T cells with a CD28^−^KLRG1^+^CD57^+^ or CD28^−^CD57^+^PD-1^+^phenotype were characterized in MM patients. Remarkably, these T cell clones showed telomere-independent senescence with upregulated telomerase activity indicating reversibility of senescence [50, 51]. Moreover, higher numbers of CD28^−^CD57^+^PD-1^+^T cells were associated with early relapse in patients with MM after autologous stem cell transplantation (ASCT) [50]. In addition, senescent and exhausted T cells in patients negatively affect T cell immunotherapy.

### Senescence and exhaustion of CAR-T cells

Currently, ACT is emerging as a potentially curative therapy for patients with advanced hematological malignancies. CAR-T and TCR-T cell therapy makes use of functionally active T cells isolated from patients. These T cells are reconstructed and expanded ex vivo to recognize specific antigens on target cells and are now widely trialed to treat leukemia, lymphoma, and several solid tumors [52–58]. However, there are functional challenges of engineered T cell therapy in regard to T cell senescence and exhaustion. Firstly, the exposure of T cells from patients to the tumor microenvironment, thus acquiring a senescent and exhausted phenotype, can lead to a progression towards terminal differentiation [59, 60]. PD-1 upregulation within the tumor microenvironment was shown to significantly inhibit T cell function indicating that CAR-T cells, which are produced from T cells with impaired function, might show less effectiveness in targeting leukemia and tumor cells [61–63]. Additionally, the endogenous TCR of T cells can have a negative influence on the persistence of CAR-T cells. Presence of TCR antigen when CAR is introduced into T cells with distinct TCR specificity was shown to provoke a loss in CD8^+^ CAR T cell efficacy associated with T cell exhaustion and apoptosis [64]. Lastly, as demonstrated by Long et al., some signaling from CAR can increase differentiation and exhaustion of T cells, in that tonic CAR CD3ζ phosphorylation, triggered by antigen-independent clustering of CAR single-chain variable fragments, will force early exhaustion of CAR-T cells [65, 66]. Overall, revision of the tumor-related T cell immune senescence and exhaustion are key points in enhancing anti-tumor function in genetically modified T cells.

### Strategies to reverse T cell senescence and restore T cell homeostasis in response to aging

**Fig. 2:**
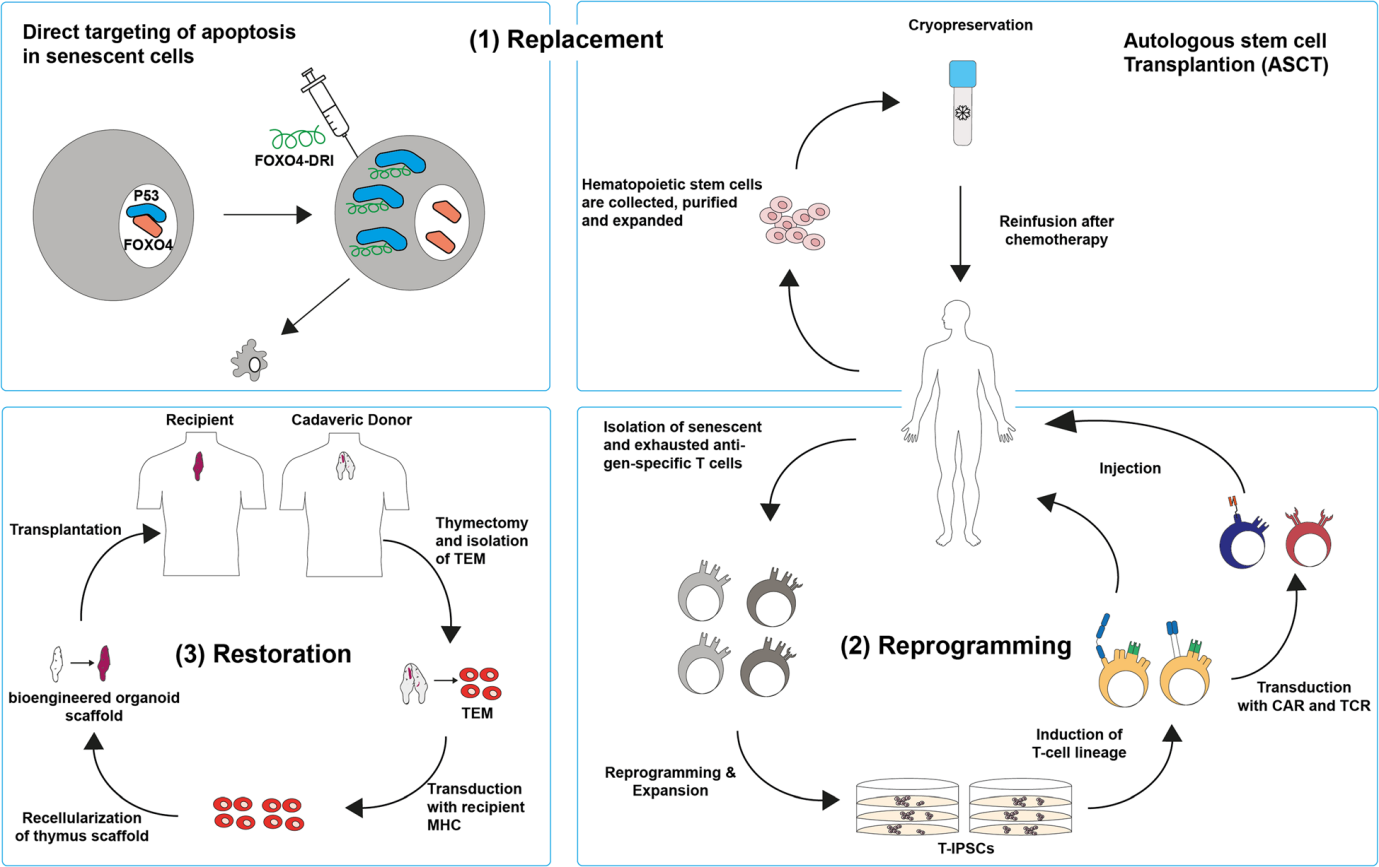
Replacement, reprogramming, and restoration strategies for reconstitution of senescent and exhausted cells in T cell pool. (1) Replacement: targeting of directed apoptosis in senescent cells as shown by Baar et al. and rebuilding the T cell pool by ASCT. Top left: blockage of P53/FOXO4 binding in fibroblasts using a bioengineered FOXO4-DRI peptide. Top right: collection, purification, and expansion of hematopoietic stem cells, followed by cryopreservation and reinfusion into patient after chemotherapy. (2) Reprogramming: isolation of senescent and exhausted antigen-specific T cells followed by reprogramming into IPSCs, expansion, induction of T cell lineage, and transduction of engineered CAR and TCR before injection. (3) Restoration: preparation of a functional thymus organoid by thymectomy of cadaveric donor, isolation of TEM, transduction with recipient MHC, and recellularization of bioengineered organoid scaffold before transplantation into recipient

There are three main strategies to rejuvenate T cell pools including replacement, reprogramming, and restoration of senescent cells. (1) Replacement strategies include the physical removal of senescent cells from the circulation with the aim of homeostatic expansion of memory and effector T cells. A possible approach is to target and promote selective apoptosis in senescent T cells. In a recent study, an engineered peptide was used to interfere with FOXO4/p53 causing targeted apoptosis in senescent fibroblasts [67], whether this also can be used in inducing apoptosis of senescent T cell remains unknown. Nevertheless, homeostatic expansion in form of autologous stem cell transplantation (ASCT) was shown to successfully reconstitute functional naïve, memory, and effector T cell pools in autoimmune diseases and hematological malignancies [68–71]. In addition, isolation and banking of cord blood HSCs has been used to reconstitute the immune system for treatment of hematological disorders and may provide hope for homeostatic expansion of functional T cells [72–74]. (2) Reprogramming is a promising method to differentiate T cells away from exhausted and senescent states by redifferentiation from T-induced pluripotent stem cells (T-IPSCs) into naive and cytotoxic T cells or dedifferentiation within their own lineage [75–77]. Although generation of T cells from human embryonic stem cells (hESCs) and iPSCs was shown to be possible, the TCR repertoire due to seemingly random VDJ gene rearrangements remains unpredictable. Nevertheless, human iPSC-derived T cells transduced with engineered TCRs and CARs specific for tumor antigens were able to infiltrate and delay tumor progression in xenograft models of solid tumors [78]. Moreover, reprogramming can potentially be used for reversion of replicative T cell senescence by enhancing telomerase activity and telomere-length restoration to extend cellular lifespan and prevent telomere-dependent T cell senescence [79, 80]. (3) Restoration strategies aim to restore and maintain the thymic environment thus reversing effects of thymic involution with help of bioengineered thymus organoids in combination with growth-promoting factors and cytokines such as IL-21, which recently was identified as a thymostimulatory cytokine and showed significant immunorestorative function and rejuvenation of the peripheral T cell pool by triggering de novo thymopoiesis in aged mice [81, 82]. Similarly, intrathymic injection of allogenic hematopoietic cells restored functional T cell development after the thymic reconstitution in a mouse model of severe combined immunodeficiency [83]. Preclinical studies have shown generation of thymic organoids from decellularized matrices as an effective approach to rejuvenate the function of T cells and the adaptive immune system. Yet, donor-specific immune tolerance, reproduction of the complex thymic extracellular matrix (ECM), and support of thymic epithelial cells, as well as T cell maturation, remain major challenges [84, 85]. Possible strategies for reversion of T cell senescence and exhaustion to restore T cell homeostasis in response to aging are summarized in Fig. 2.

### Targets for recovery of T cell activation in hematological malignancies

**Fig. 3:**
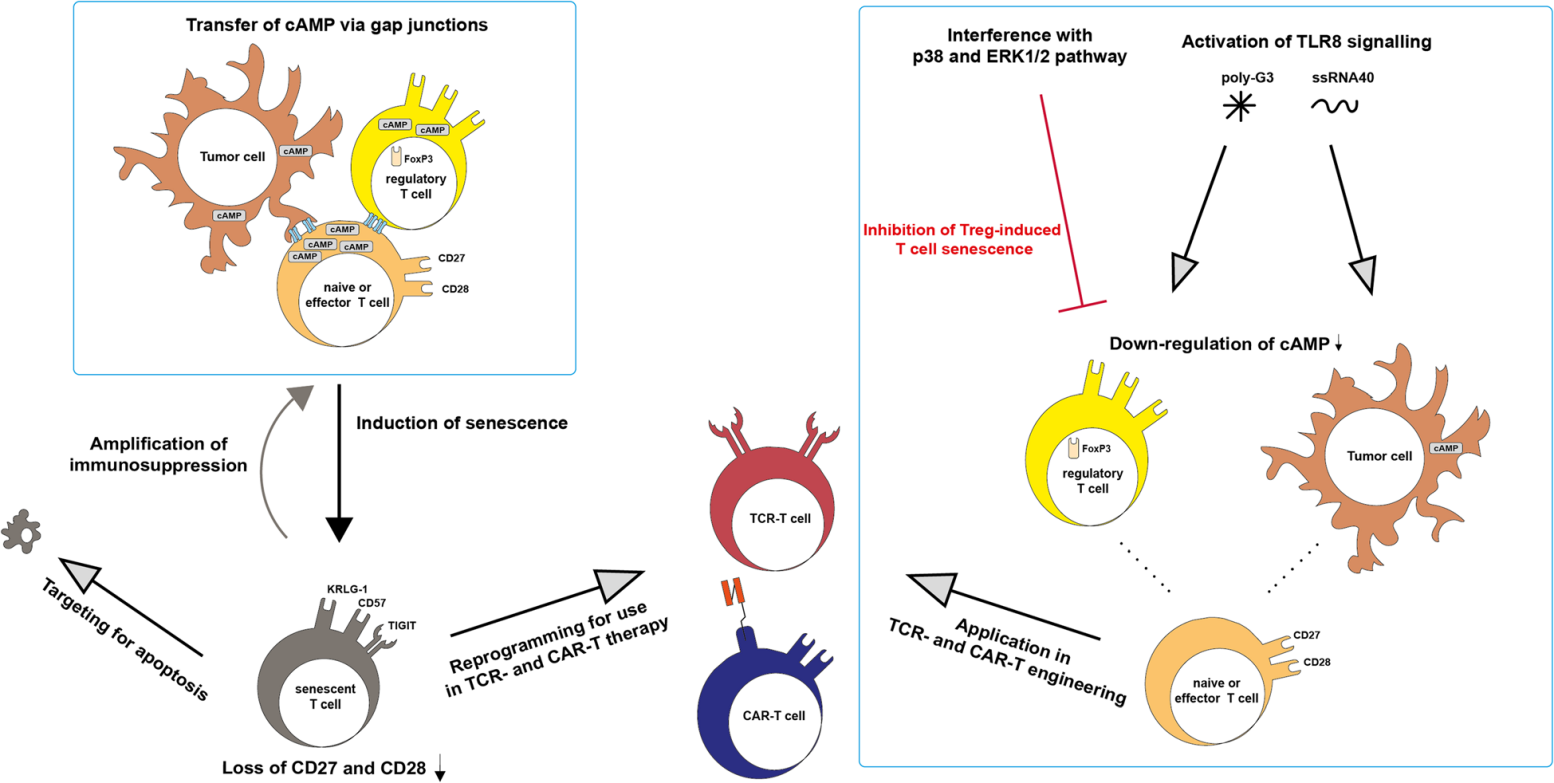
Mechanism of T cell senescence induction in the tumor microenvironment and strategies for revision of T cell senescence for TCR- and CAR-T cell therapy. Treg and tumor cells use cAMP as a key component in senescence induction of naive and effector T cells. Induction of senescence in T cells leads to a loss of CD27 and CD28 and an amplification of immunosuppression in the tumor microenvironment. Interference with p38 and ERK1/2 as well as activation of TLR8 signaling by poly-G3 and ssRNA40 downregulates cAMP levels in Treg and tumor cells, disrupting induction of senescence and immunosuppression. Functional T cells then can be further used in adoptive immunotherapies. Alternatively, senescent T cells can be reprogrammed for dedifferentiation to use in TCR- and CAR-T cell therapies or targeted for apoptosis to help to recover the homeostasis of T cell subsets in patients with hematological malignancies

Tumor sites in hematological malignancies were shown to recruit Treg cells and use direct and indirect induction of senescence in their tumor microenvironments as a mechanism of immune suppression [86–89, 48, 49]. Therefore, one possible target of immunotherapy is the inhibition of tumor-related T cell senescence as well as the possible restoration of senescent T cell function. In cases of increased numbers of CD28^−^CD57^+^PD-1^+^T cells in MM patients, PD-1 blockade was shown to restore proliferation and cytokine secretion in exhausted/senescent CD8+ T cells [87]. Elevated levels of transcription factor B lymphocyte-induced maturation protein 1 (Blimp-1) in patients suffering from AML correlated with upregulation of multiple inhibitory receptors including PD-1 and TIGIT on exhausted, functionally impaired T cells. More importantly, siRNA knockdown of Blimp-1 has shown to reverse the functional defect [90]. cAMP, which is also a key component of Treg cell suppression in aging, is accumulated in tumor sites creating hypoxic microenvironments. Treg and tumor cells in these microenvironments directly induced human naïve T cells and tumor-specific effector T cells to become senescent by increasing cAMP levels using transfer via gap junctions [91, 92]. Due to their inherent suppressive function, dysfunctional senescent T cells then can indirectly maintain the tumor microenvironment and amplify immunosuppression [93], thus indicating that regulation of cAMP level might be a potential approach to revise T cell senescence and disrupt the tumor microenvironment in patients. Recent studies implicate metabolic regulation of tumor cells by Toll-like receptor 8 (TLR8) signaling. Specifically, TLR8 ligands, such as third-generation polyamidoamine dendrimers (poly-G3) and ssRNA40, were shown to enhance antitumor immunity by modulation of endogenous cAMP in tumor cells through the activation of the protein kinase A (PKA) type I–COOH-terminal Src kinase (Csk)–LCK inhibitory pathway [94]. Moreover, ERK1/2 and P38 signaling was identified as regulators of Treg-induced senescent T cells [19, 25]. These results open a possibility to reverse the suppression by tumor microenvironments, creating effector microenvironments by modulation of specific factors in tumor-related T cell senescence. Interestingly, a common alteration in childhood T cell acute lymphoblastic leukemia (T-ALL) cells is the deletion of p16 and p15 and in some cases hypermethylation of a 5′ CpG island in the p15 gene. The accumulation of both proteins is strongly associated with T cell aging and senescence, and thus, their deletion might indicate a role in immortalization and the mechanism of senescence avoidance by some leukemic T cells [24]. Although this indicates that interference with the accumulation of p16 can possibly slow down aging or prevent senescence of T cells, it also potentially harbors an increased risk of provoking T cells to become cancerous and hence should be explored further. Mechanisms of T cell senescence induction in the tumor microenvironment and strategies for revision of T cell senescence for TCR- and CAR-T cell therapy are shown in Fig. 3.

To improve persistence and effectiveness of CAR-T cells, it is necessary to establish assays to characterize the T cell status in patients who are selected for CAR-T cell therapy. Next, depending on the immune alterations in these patients, different targeting approaches can be chosen to revise senescence and exhaustion as depicted in Figs. 2 and 3. Finally, therapies including PD-1 checkpoint blockade, which can overcome the immune evasion of tumor cells from CAR-T cells within the tumor microenvironment, and the use of apoptosis inhibitor blockade agents, to increase the effect of CAR-T cell therapy, can significantly improve CAR-T cell effectiveness [58, 61, 95, 96].

## Conclusion

T cell senescence is playing a key role in immune suppression and evasion of both hematological and solid tumors. Understanding the underlying mechanisms of Treg cell recruitment as well as direct and indirect induction of T cell senescence by tumor microenvironments will open new immunotherapeutic strategies for restoration and recovery of TCR-T and CAR-T cell activation after senescence and exhaustion. Specifically, replacement, reprogramming, and restoration of the immune system as well as modulation of signaling in tumor sites, shifting immunosuppressive microenvironments to become effector microenvironments, are promising approaches. Further, occurrence of potentially reversible telomere-length independent senescent T cells in hematological malignancies has to be investigated more extensively. Understanding of its occurrence might potentially give insight into reversion of replicative T cell senescence for optimized CAR-T or TCR-T cell immunotherapy.

## Abbreviations

ACT: Adoptive cellular immunotherapy
ALL: Acute lymphoblastic leukemia
AML: Acute myeloid leukemia
ASCT: Autologous stem cell transplantation
Blimp-1: B lymphocyte-induced maturation protein 1
cAMP: Cyclin adenosine monophosphate
CAR-T: Chimeric antigen receptor expressing T cell
CD: Cluster of differentiation
CLL: Chronic lymphocytic leukemia
CTLA4: Cytotoxic T-lymphocyte-associated protein 4
FOXO4: Forkhead box protein O4
HCMV: Human cytomegalovirus
hTERC: Human telomerase RNA component
IDO: Indoleamine-pyrrole 2,3-dioxygenase
IL-10: Interleukin-10
ITIM: Immunoreceptor tyrosine-based inhibitory motif
KLRG-1: Killer cell lectin-like receptor sub family G
LAG3: Lymphocyte-activation protein 3
LFS: Leukemia-free survival
MAPK: Mitogen-activated protein kinase
MM: Multiple myeloma
PD-L1: Programmed cell death ligand 1
PKA: Protein kinase A
poly-G3: Third-generation poly amidoamine dendrimers
TCR: T cell receptor
TGF-β: Transforming growth factor beta
TIGIT: T cell immunoreceptor with Ig and tyrosine-based inhibitory motif (ITIM) domains
TIM3: T cell immunoglobulin domain and mucin domain-containing protein3
T-IPSCs: T-induced pluripotent stem cells
TLR8: Toll-like receptor 8
Treg: Regulatory T
